# Social withdrawal and academic achievement, intertwined over years? Bidirectional effects from primary to upper secondary school

**DOI:** 10.1111/bjep.12504

**Published:** 2022-04-06

**Authors:** Frode Stenseng, Eivind B. Tingstad, Lars Wichstrøm, Vera Skalicka

**Affiliations:** ^1^ Department of Education and Lifelong Learning Norwegian University of Science and Technology Trondheim Norway; ^2^ Oslo New University College Oslo Norway; ^3^ Department of Psychology Norwegian University of Science and Technology Trondheim Norway

**Keywords:** belongingness, peer interaction, school performance, self‐esteem, social competence

## Abstract

**Background:**

Socially withdrawn children tend to perform poorer academically than their peers. What remains unknown, is the temporal ordering of the two phenomena. Also, substantial gender differences exist in both social withdrawal and academic achievement; thus, it is conceivable that the strength of the relation between them is gendered as well.

**Aims:**

To investigate cross‐sectional correlates and test directional effects of social withdrawal and academic achievement from primary to upper secondary school, and to examine potential gendered effects.

**Methods:**

Prospective associations were analysed from age 6 to age 14 using biannual teacher ratings of children's social withdrawal and academic achievement in a representative community sample (*n *= 845), by means of random intercept cross‐lagged panel modelling.

**Results:**

In boys, increased academic achievement at ages 8 and 12 forecasted decreased social withdrawal 2 years later, whereas increased social withdrawal at age 10 predicted reduced academic achievement at age 12. No such effects were seen in girls.

**Conclusions:**

Social withdrawal and academic achievement are bidirectionally related among boys, but not girls. Results are discussed in light of need‐to‐belong theory, and practical implications for schools and teachers are illuminated.

## BACKGROUND

Social withdrawal is rooted in various causes ranging from a volitional drive for solitude to pacifying social inhibition (Rubin & Asendorpf, 1993; Rubin et al., 2003). In cases of the latter type of social withdrawal, often defined as *conflicted shyness* (Rubin, Copland, & Bowker, 2014), the situation obstructs the child's pathway to desired social connections. The prevalence of problematic social withdrawal varies across cultures, but some studies estimate it to be 5%–10% (e.g. Wu et al., 2020), and overlaps with other problematic social factors, such as social anxiety and increased risk of victimization (for a review, see Rubin & Chronis‐Tuscano, 2021), as well as poorer language skills (Rudasill et al., 2014).

According to the need‐to‐belong theory (Baumeister & Leary, 1995), humans have a fundamental motivation to create and maintain long‐term relationships. When failing to establish such relationships, the need for *belongingness* becomes thwarted and a stressful psychological state emerges which burdens cognitive and regulatory resources. (Baumeister et al., 2007). Conflicted shyness (Rubin, Copland, & Bowker, 2014) is characterized by behaviour where the child is socially alert and motivated for social participation but fails to take active part in other children's dialogues and activities. When children experience this kind of internal approach–avoidance conflict, it obstructs their fulfilment of their need for belongingness, and consequences like those originating from social rejection may emerge. Extensive research has been conducted on the outcome of social rejection and exclusion. Several experimental studies have found that social exclusion may cause impaired attention regulation, reduced persistence on difficult tasks and poorer results in cognitive tests, such as IQ tests (Baumeister et al., 2002, 2005). Longitudinal community studies have found that long‐term effects of social exclusion in childhood include impaired trait self‐esteem (Harris & Orth, 2020), more aggressive behaviour and more ADHD symptoms (Stenseng, Belsky, Skalicka, & Wichstrøm, 2014, 2015, 2016). Coinciding with these findings, several studies have shown that social withdrawal in childhood increases the risk of psychosocial maladjustment and mental health problems, such as depression, loneliness and anxiety (e.g. Coplan et al., 2004). It is well established that dejection emotions, such as those related to dysthymia or depression, dampen the cognitive processes necessary for optimal learning and achievement (i.e. attentional focus) (e.g. Pekrun et al., 2002). As shown in a short‐term longitudinal study by Buhs and Ladd (2001) conducted among 5‐year‐olds, preschool teachers reported those who experienced more peer rejection in the fall semester to show more negative affect in spring, as well as lower academic performance and decreased classroom participation. Accordingly, Hall et al. (2016) found that higher levels of social withdrawal in kindergarten predicted lower reading abilities at second grade in primary school, controlled for kindergarten literacy.

In a broader context, a recent meta‐analysis by Korpershoek et al. (2020) concluded that higher levels of school belonging (including social bonds) in secondary education are correlated with a range of favourable educational outcomes, including more adaptive motivation, engagement and academic achievement. This concurs with Eisenberg et al. (1998), who suggested that reserved behaviour in the classroom obstructs students’ leeway to learning because classroom participation, collective proficiency and social adjustment are prerequisites for academic development (see also Hall et al., 2016). Accordingly, deprivation of belongingness in the school context seems to undermine the roots of academic accomplishment, whereas satisfaction with it seems to fuel academic development.

With regard to the opposite causal direction that higher academic achievement may facilitate better social functioning, arguments for such a relationship may be found in self‐esteem literature. As noted by Rosenberg et al. (1995), general self‐esteem is distinguishable from specific self‐esteem, so self‐esteem may be differentiated in separate life areas, and tied to one specific social domain, such as school. This is in line with a review by Baumeister et al. (2003), suggesting that higher general self‐esteem may be viewed as an outcome of academic performance rather than a cause, illustrating that self‐esteem in one domain may be transmitted to the broader self. Thus, there is reason to believe that increased achievement in the school domain may be transmitted to global self‐esteem, which manifests as more outgoing social behaviour, at least in the school/class environment. Such a *crossover effect* of self‐esteem (see also Stenseng & Dalskau, 2010; Pierce et al., 2016) may spur cascades in which higher academic achievement results in less social withdrawal in the school setting, which might in turn foster more favourable academic development.

Girls and boys differ in their levels of academic maturity throughout childhood and adolescence (Pomerantz et al., 2002); thus, one may expect gender to moderate how the above‐mentioned mechanisms unfold. However, the extent to which the outcomes of social withdrawal are gendered has been debated, and the results are mixed. In a review by Doey et al. (2014), it was suggested that social withdrawal constitutes a greater risk for boys than girls in terms of developmental problems. Building on this review, Rubin and Barstead (2014) conducted specific analyses of their data from *The Friendship Project*, finding that withdrawn 10‐ to 12‐year‐old boys were more likely than girls to be excluded and victimized by their peers.

Based on gender stereotypes, it could also be argued that shyness among boys makes them more vulnerable to negative teacher evaluations than among girls. Introversion normally diverges from masculinity traits (e.g. seeking power, showing strength; see Kling et al., 1999) so that reclusive behaviour may negatively affect how teachers perceive withdrawn boys’ academic engagement. However, since it is more phenotypical for girls to perform well in school, an increase in boys’ academic performance may be more socially facilitating for boys than for girls, for whom such academic performance might be less anticipated.

From the above‐mentioned literature (i.e. Baumeister & Leary, 1995), it seems plausible to suggest that social withdrawal and academic achievement are intertwined throughout childhood and adolescence; however, prospective studies testing these potential cross‐directional and reciprocal effects are lacking. Moreover, we expected that increased social withdrawal would predict decreased academic performance over time and that improved academic performance would predict decreased social withdrawal between time points. Based on previous studies, we hypothesized that these effects would be most pronounced in boys. Hence, we tested the bidirectional relationship between social withdrawal and academic achievement using a five‐wave prospective sample. Multi‐group analyses were conducted to analyse gender differences, and cross‐sectional overlaps were also analysed. Because academic achievement and social functioning are rooted in common underlying causes, such as genetics, parental SES, cognitive abilities, temperament and parenting (Bartels et al., 2002; Kurdek & Sinclair, 2000), we applied random intercept cross‐lagged panel modelling which accounts for all such unmeasured time‐invariant confounders while illuminating paths at the within‐person level.

## METHOD

### Participants and procedure

**FIGURE 1:**
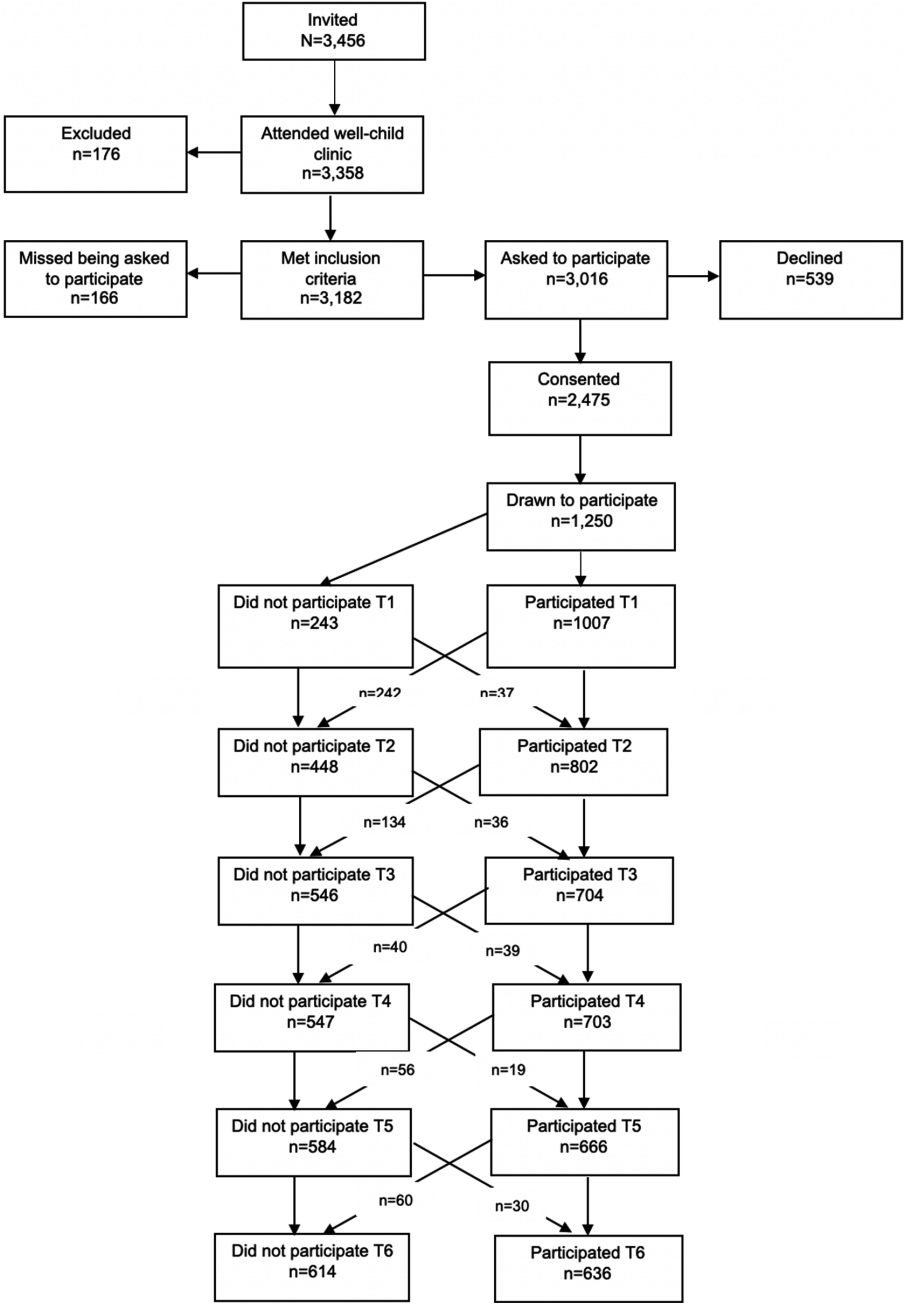
Procedure and flow of participants in the Trondheim Early Secure Study

The first wave of the Trondheim Early Secure Study (TESS; Steinsbekk & Wichstrøm, 2018) took place in 2007 and 2008 (T1) and included children born in 2003 or 2004 with parents living in Trondheim, Norway. This study used data from the second through sixth waves of data collection, when the children were 6, 8, 10, 12 and 14 years old, attending the first, third, fifth, seventh and ninth grades of elementary school respectively. A total of 1250 children were recruited to participate in the study, of which 997 were tested at the time of the study enrolment (*M*_age_ = 4.55 years; 50.6% boys). At T1, 81% of the children were accompanied by their mothers, more than 99% of the children were of Western ethnic origin (e.g. Europe, United States), and 86% of these children had parents who lived together. Further reading of the procedure, recruitment and sample has been presented elsewhere (Steinsbekk & Wichstrøm, 2018). Figure 1 shows the participation rates and flow of the participants in more detail.

Teacher data were collected through questionnaires sent to schools and colleges. Teachers provided information on social withdrawal and academic achievement at all measurement points. Response rates among teachers were as follows: age 6 = 94.1%, age 8 = 92.2%, age 10 = 85.8%, age 12 = 82.3% and age 14 = 80.7%. This project was approved by the Regional Committee for Research Ethics, Mid‐Norway (www.etikkom.no; REK 4.2008.2632).

### Measures

#### Social withdrawal

The Child Social Preference Scale (CSPS) (Coplan et al., 2004) was developed to assess social withdrawal (SW) among children. The CSPS instrument was originally a two‐dimensional scale containing the dimensions of Shyness and Social Disinterest. In the TESS data, only the Conflicted Shyness dimension is available. Teachers rated each child on the dimension, consisting of seven items, with sample items such as ‘*The child declines social initiatives from other children because he*/*she is shy’* and ‘*The child circles other children's play without participating’*. Teachers in Norway normally engage in social activities outside the classroom, and co‐learning is a widely used pedagogical tool that adds validity to these scores. Teachers rated each child for each item on a 5‐point Likert scale, ranging from 0 (*to a small degree*) to 5 (*to a large degree*). The total of these responses was computed as the sum score for each child (see Table 1). Acceptable internal consistency coefficients were found for the construct, with Cronbach's alphas ranging from .87 to .76 over measure points.

**TABLE 1:** Means, standard deviations and bivariate correlations for study variables

	*M*	*SD*	1	2	3	4	5	6	7	8	9	10	11
1. Gender	‐	‐	‐										
2. Academic achievement, age 6	9.81	2.31	.12**	‐									
3. Social withdrawal, age 6	10.41	4.36	.00	−.17**	‐								
4. Academic achievement, age 8	9.95	2.54	.16**	.69**	−.14*	‐							
5. Social withdrawal, age 8	9.58	3.58	−.01	−.12*	.47**	−.15**	‐						
6. Academic achievement, age 10	9.83	2.64	.11**	.62**	−.14**	.74**	−.14**	‐					
7. Social withdrawal, age 10	10.52	4.41	.02	−.18**	.38**	−.22**	.37**	−.26**	‐				
8. Academic achievement, age 12	10.04	2.52	.15**	.54**	−.15**	.66**	−.15**	.77**	−.19**	‐			
9. Social withdrawal, age 12	10.44	4.71	−.04	−.17**	.30**	−.15**	.39**	−.09*	.50**	−.24**	‐		
10. Academic achievement, age 14	9.99	2.03	.14**	.49**	−.12*	.56**	.22**	.62**	−.20**	.69**	−.19**	‐	
11. Social withdrawal, age 14	11.78	5.23	−.02	−.18**	.24**	−.16**	.29**	−.19**	.35**	−.22**	.48**	−.24**	‐

* *p* < .05.

** *p* < .01.

#### Academic achievement

The Teacher Report Form (TRF) from the Achenbach System of Empirically Based Assessment (ASEBA) (Achenbach & Rescorla, 2000) was used to assess academic achievement (AA). Teachers reported the children's proficiency in three school subjects: reading, writing and mathematics. Teachers rated each of these academic proficiencies for each child on a 5‐point scale, 1 (*far below grade*), 2 (*somewhat below grade*), 3 (*at grade level*), 4 (*somewhat above grade*) or 5 (*far above grade*), with a maximum score of 15 and a minimum score of 3 (see Table 1). Teacher ratings of the children reflect the long‐term development of their academic achievements in the classroom, as opposed to standardized tests that provide an assessment of the child's competence at a single point in time. The sum of the scores of these assessments (math, reading and writing) was computed for each time point. High internal consistency coefficients were found for the construct, with Cronbach's alpha values ranging from .76 to .89.

### Data analysis

Cross‐lagged effects of social withdrawal and academic performance from ages 6 to 14 were examined using structural equation modelling (SEM) in M*plus* 8.1 (Muthén & Muthén, 2017). A random intercept cross‐lagged panel model (RI‐CPLM) was tested (Hamaker et al., 2015). This model refines the traditional autoregressive cross‐lagged model of reciprocal relations between SW and AA by adding a stable *between*‐*person* component (represented by two latent random intercepts loading on observed measures of AA and SW, respectively, across time) and a latent *within*‐*person* component, which assesses changes in one's own mean level (e.g. AA_t3_) as a function of changes in one's own levels at a previous time point (e.g. AA_t2_ and SWt_2_, autoregressive and cross‐lagged effects). Missing values were treated using the full information maximum‐likelihood estimator (FIML). Multi‐group analyses by sex were evaluated using the Satorra–Bentler Chi‐square test (Satorra & Bentler, 2001). Model fit was determined according to the recommendations of Hu and Bentler (1998).

## RESULTS

Weighted descriptive analyses, including mean‐level differences between measurement times and bivariate correlations from M*plus* 8.1, are presented in Table 1.

A RI‐CLPM fitted the data well (χ^2^ (21) = 35.759, *p* = .02, RMSEA = .029, SRMR = .034, CFI = .992, TLI = .982). At the between‐person level, children with higher AA manifested less SW (*r* = −.34, 95% CI: −.49 to −.19). At the within‐person level, no significant prospective cross‐lagged associations were detected between AA and SW.

**FIGURE 2:**
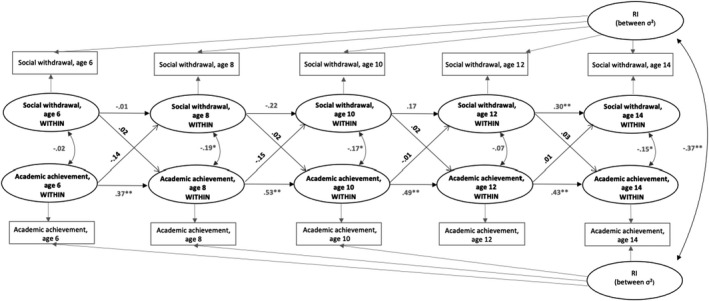
Within‐subjects cross‐lagged effects of Social Withdrawal and Academic Achievement from random intercepts modelling for girls, ages 6, 8, 10, 12 and 14. Path coefficients are standardized regression weights (*p < .05, **p < .01 level)

**FIGURE 3:**
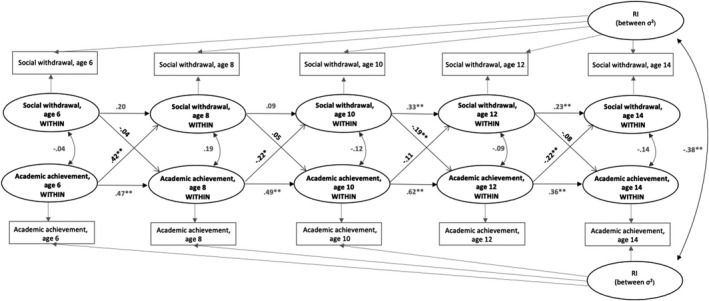
Within‐subjects cross‐lagged effects of Social Withdrawal and Academic Achievement from random intercepts modelling for boys, ages 6, 8, 10, 12 and 14. Path coefficients are standardized regression weights (*p < .05, **p < .01 level)

Multi‐group analyses for each gender revealed that among boys at the within‐person level, more SW at age 10 predicted decreased AA from age 10 to age 12 (*β* = −.19, *p* = .003). However, these effects were not significantly different from the respective non‐significant paths at other ages (Δχ^2^ = 10.06 [7], *p* = .19). In contrast, the effects of boys’ AA on later SW varied across age groups (Δχ^2^ = 29.2 [7], *p* < .001). Boys’ AA at age 8 predicted decreased SW from age 8 to age 10 (*β* = −.22, *p* = .04), and a similar effect was observed for higher AA at age 12, predicting attenuated SW from age 12 to age 14 (*β* = −.22, *p* = .005). Inconsistent with these results, boys’ AA at age 6 predicted higher SW at age 8 (*β* = .42, *p* = .002). None of these effects was significant in girls (Figures 2 and 3). The gender difference proved significant in SW 10 → AA 12 (Δχ^2^ = 7.24 [1], *p* = .007), AA 6 → SW 8 (Δχ^2^ = 18.70 [1], *p* < .001) and AA 12 → SW 14 (Δχ^2^ = 4.86 [1], *p* = .028).

## DISCUSSION

The primary aim of this study was to examine the prospective relationship between social withdrawal and academic achievement for schoolchildren across an 8‐year period, from the first grade (age 6) to the ninth grade (age 14). Bidirectional effects of this interplay were proposed: (1) increased social withdrawal predicts decreased academic performance and (2) improved academic performance promotes lessened social withdrawal (over biannual measurements).

First, and cross‐sectionally, we found that social withdrawal and academic achievement were negatively related at all five measurement points. Of note, such a relationship has been found in several cross‐sectional studies (e.g. Coplan et al., 2001; Crozier, 1995; Fantuzzo et al., 2003; Wentzel, 1991; Wentzel & Caldwell, 1997). Many mechanisms may explain this negative association. For example, because of the interactive and social nature of learning, socially withdrawn children might have more time to acquire the learning skills necessary for successful engagement in the classroom (Domínguez et al., 2010). Academic engagements, such as oral presentations, group projects and oral activity/classroom discussions, may prove challenging for children inhibited by social anxiety or fear of expressing themselves. When children avoid peer interaction, they are at risk of showing a lack of academic engagement and participation in school, which may dwindle their teachers’ ratings of their academic performance (Hughes & Coplan, 2010). In addition, there are numerous potential spurious effects that cause both high social withdrawal and low academic achievement simultaneously, conceivably rooted in children's temperament and/or cognitive abilities.

Second, cross‐lagged longitudinal analyses controlled for between‐person variance and projected effects among boys but not among girls. For boys, higher academic achievement at age 8 attenuated social withdrawal from ages 8 to 10, and this was repeated from ages 12 to 14. In addition, greater social withdrawal at age 10 predicted lower academic achievement from 10 to 12 years of age. These results indicate that cascade‐like relations may emerge for some boys; better academic achievement results in less social withdrawal, which then supports more optimal academic development, or antagonistically, lower academic performance instigates more social withdrawal, which then leads to lower academic performance. These gendered effects are relevant to the findings of Doye et al. (2014) and the follow‐up analyses made by Rubin and Barstead (2014), supporting the notion that withdrawn boys are more vulnerable to negative social effects than girls. Potential explanations for these gender effects are indicated in the introduction; being outgoing may be more valuable for boys than girls, because it is associated with masculinity, and thus social withdrawal has more fundamental negative consequences on academic achievement for boys than girls, perhaps especially during puberty (Kling et al., 1999). This could explain how socially withdrawn boys were perceived by their teachers. For instance, socially withdrawn boys tend to perform poorly on oral examinations in school, thus risking lower grades (Crozier & Hostettler, 2003). This is underscored by Hughes and Coplan (2010), who found that child shyness correlated more negatively with teacher‐rated academic performance than scores on standardized scholastic tests. The gender effect in our data could also originate from internal psychological processes among boys such as changes in self‐esteem. Large community studies have consistently found that adolescent boys – in general – score higher than girls on self‐esteem (Kling et al., 1999). As reported by von Soest et al. (2016), from a large Norwegian prospective community study looking at six domains of specific self‐esteem, boys scored lower than girls in two domains: social acceptance and close relationships. Thus, the observed crossover effect of academic achievement on less social withdrawal observed only among boys may be due to the greater potential for upward change among boys when it comes to social self‐esteem and social functioning. Marino et al. (2020) found that levels of self‐esteem were modifiable through a mentoring programme (Mentor‐UP) conducted in Italian schools among 11‐to 13‐year‐olds, indicating that tailored interventions aimed at enhancing self‐esteem in schools may constitute a path for building better academic performance. Potentially, such programmes may be most favourable for boys who are high in social withdrawal but low in self‐esteem.

As mentioned, the fundamental need to achieve social acceptance and inclusion is at the core of the need‐to‐belong theory (Baumeister & Leary, 1995). Previous longitudinal studies found that social exclusion in the school context compromises the development of self‐regulation (Stenseng et al., 2015, 2016), which is further linked to adaptive learning strategies. An interpretation of the present results that increased academic achievement leads to less social withdrawal is that students who perform well in school are likely to be perceived as successful by their peers, which make them more attractive as learning partners, and enhances their social status among peers. This could further accelerate their interest in and motivation to interact with co‐students and teachers (Wentzel & Caldwell, 1997). A cycle of positive events could occur: the cycle begins with an increase in academic achievement, which boosts students’ self‐esteem, social status and motivation for peer interactions. This increase in self‐esteem and social interaction could reduce children's levels of shyness and improve their self‐regulation and learning skills, which affect both social and academic functioning at school.

### Limitations

This study adds to the literature on the academic achievement of socially withdrawn children, but some limitations in this study must be discussed. First, this study did not consider potential time‐varying factors, such as family, classroom, peer and teacher characteristics that could influence the academic performance of socially withdrawn children. Second, although the internal consistency of the academic domains (math, reading and writing) was high across the respective school years, there is a risk that a differentiated pattern of relationships is masked when using computed sum scores. However, although domain‐specific hypotheses were not investigated in this study, they may be warranted in future research. Third, this study only assessed the conflicted shyness dimension of the Child Social Preference Scale (Coplan et al., 2004), leaving out two other dimensions: social disinterest and active isolation. It is conceivable that these dimensions of social withdrawal may relate differently to academic performance than is projected in this study. Fourth, whereas teachers may have a rather solid footing for rating their students’ level of social withdrawal in the early school years, which is a time when much of the learning in and outside the classroom is play based, this may diminish as the children enter the secondary school years, when teaching becomes more structured and less improvised the latter is also true socially. Finally, we are short of a meaningful interpretation of the positive effect of academic achievement at age 6 on social withdrawal at age 8, controlled for age 6 levels, found among boys. One suggestion is that academically mature boys steer away from play activities typical for boys at this age, which then is misinterpreted as social withdrawal; however, this clearly needs further exploration.

### Implications and conclusions

As shown in this study, social withdrawal is entangled with academic achievement both cross‐sectionally and prospectively. Our findings show that academic performance promotes social functioning, and vice versa, but primarily among boys, which is in accordance with previous research showing that boys are more vulnerable to social withdrawal than girls (Doyle et al, 2014). Traditionally, at least in Western schools, the emphasis has been on creating a safe and nurturing social context for withdrawn children, which is supposed to instigate optimal learning. Specifically, efforts to attenuate social withdrawal have mostly been rooted in children's social preconditions. However, this study showed that higher academic performance by itself may promote better social functioning. In conclusion, the present findings provide valuable additions to the literature on the relationship between social withdrawal and academic achievement through the elementary school years. These findings highlight the importance of the teacher's awareness for optimal learning conditions for withdrawn children, which may include learning environments with fewer participants, more flexible dates and contexts for assignments and presentations, and more supportive and consistent student–teacher communication (see Kalutskaya et al., 2015). Future studies may aim to develop successful interventions which promote academic performance among socially withdrawn children in particular, and developing tools aimed at increasing academic self‐esteem may be a promising path (e.g. Marino et al., 2020).

## CONFLICT OF INTEREST

All authors declare no conflict of interest.

## AUTHOR CONTRIBUTION

**Frode Stenseng:** Conceptualization; Data curation; Formal analysis; Funding acquisition; Investigation; Methodology; Project administration; Visualization; Writing – original draft; Writing – review & editing. **Lars Wichstrøm:** Funding acquisition; Investigation; Methodology; Project administration; Writing – original draft; Writing – review & editing. **Eivind Tingstad:** Conceptualization; Project administration; Writing – original draft; Writing – review & editing. **Vera Skalicka:** Conceptualization; Data curation; Formal analysis; Methodology; Writing – original draft; Writing – review & editing.

## Data Availability

The prospective data used this study are available on request from the corresponding author. The data are not publicly available due to privacy and/or ethical restrictions.

## References

[bjep12504-bib-0001] Achenbach, T. M. , & Rescorla, L. A. (2000). Manual for the ASEBA preschool forms & profiles: An integrated system of multi‐informant assessment; Child Behavior Checklist for ages 1 ½–5. Language development survey; caregiver‐ teacher report form. Burlington: University of Vermont.

[bjep12504-bib-0002] Bartels, M. , Rietveld, M. J. , Van Baal, G. C. M. , & Boomsma, D. I. (2002). Heritability of educational achievement in 12‐year‐olds and the overlap with cognitive ability. Twin Research, 5(6), 544–553. 10.1375/136905202762342017 12573186

[bjep12504-bib-0003] Baumeister, R. F. , Brewer, L. E. , Tice, D. M. , & Twenge, J. M. (2007). Thwarting the need to belong: Understanding the interpersonal and inner effects of social exclusion. Social and Personality Psychology Compass, 1(1), 506–520. 10.1111/j.1751-9004.2007.00020.x

[bjep12504-bib-0004] Baumeister, R. F. , Campbell, J. D. , Krueger, J. I. , & Vohs, K. D. (2003). Does high self‐esteem cause better performance, interpersonal success, happiness, or healthier lifestyles? Psychological Science in the Public Interest, 4(1), 1–44. 10.1111/1529-1006.01431 26151640

[bjep12504-bib-0005] Baumeister, R. F. , DeWall, C. N. , Ciarocco, N. J. , & Twenge, J. M. (2005). Social exclusion impairs self‐regulation. Journal of Personality and Social Psychology, 88(4), 589–604. 10.1037/0022-3514.88.4.589 15796662

[bjep12504-bib-0006] Baumeister, R. F. , & Leary, M. R. (1995). The need to belong: Desire for interpersonal attachments as a fundamental human motivation. Psychological Bulletin, 117(3), 497–529. 10.1037/0033-2909.117.3.497 7777651

[bjep12504-bib-0007] Baumeister, R. F. , Twenge, J. M. , & Nuss, C. K. (2002). Effects of social exclusion on cognitive processes: Anticipated aloneness reduces intelligent thought. Journal of Personality and Social Psychology, 83(4), 817–827. 10.1037/0022-3514.83.4.817 12374437

[bjep12504-bib-0046] Buhs, E. S. , & Ladd, G. W. (2001). Peer rejection as antecedent of young children's school adjustment: An examination of mediating processes. Developmental Psychology, 37(4), 550–560. 10.1037/0012-1649.37.4.550 11444490

[bjep12504-bib-0008] Coplan, R. J. , Gavinski‐Molina, M. H. , Lagacé‐Séguin, D. G. , & Wichmann, C. (2001). When girls versus boys play alone: Nonsocial play and adjustment in kindergarten. Developmental Psychology, 37(4), 464–474. 10.1037/0012-1649.37.4.464 11444483

[bjep12504-bib-0009] Coplan, R. J. , Prakash, K. , O’Neil, K. , & Armer, M. (2004). Do you “want” to play? Distinguishing between conflicted shyness and social disinterest in early childhood. Developmental Psychology, 40(2), 244–258. 10.1037/0012-1649.40.2.244 14979764

[bjep12504-bib-0010] Crozier, W. R. (1995). Shyness and self‐esteem in middle childhood. British Journal of Educational Psychology, 65(1), 85–95. 10.1111/j.2044-8279.1995.tb01133.x 7727270

[bjep12504-bib-0011] Crozier, W. R. , & Hostettler, K. (2003). The influence of shyness on children’s test performance. British Journal of Educational Psychology, 73(3), 317–328. 10.1348/000709903322275858 14672146

[bjep12504-bib-0048] Doey, L. , Coplan, R. J. , & Kingsbury, M. (2014). Bashful boys and coy girls: A review of gender differences in childhood shyness. Sex Roles, 70, 255–266. 10.1007/s11199-013-0317-9

[bjep12504-bib-0045] Domínguez, X. , Vitiello, V. E. , Maier, M. F. , & Greenfield, D. B. (2010). A longitudinal examination of young children’s learning behavior: Child‐level and classroom‐level predictors of change throughout the preschool year. School Psychology Review, 39(1), 29–47. 10.1080/02796015.2010.12087788

[bjep12504-bib-0013] Eisenberg, N. , Shepard, S. A. , Fabes, R. A. , Murphy, B. C. , & Guthrie, I. K. (1998). Shyness and children’s emotionality, regulation, and coping: Contemporaneous, longitudinal, and across‐context relations. Child Development, 69(3), 767–790. 10.1111/j.1467-8624.1998.00767.x 9680684

[bjep12504-bib-0014] Fantuzzo, J. N. , Bulotsky, R. , McDermott, P. , Mosca, S. , & Lutz, M. N. (2003). A multivariate analysis of emotional and behavioral adjustment and preschool educational outcomes. School Psychology Review, 32(2), 185–203. 10.1080/02796015.2003.12086193

[bjep12504-bib-0015] Hall, C. M. , Welsh, J. A. , Bierman, K. L. , & Nix, R. (2016). Kindergarten social withdrawal and reading achievement: A cross‐lagged path model for at‐risk learners. Psychology in the Schools, 53(7), 751–759. 10.1002/pits.21939

[bjep12504-bib-0016] Hamaker, E. L. , Kuiper, R. M. , & Grasman, R. P. (2015). A critique of the cross‐lagged panel model. Psychological Methods, 20(1), 102–116. 10.1037/a0038889 25822208

[bjep12504-bib-0017] Harris, M. A. , & Orth, U. (2020). The link between self‐esteem and social relationships: A meta‐analysis of longitudinal studies. Journal of Personality and Social Psychology, 119(6), 1459–1477. 10.1037/pspp0000265 31556680

[bjep12504-bib-0018] Hu, L.‐T. , & Bentler, P. M. (1998). Fit indices in covariance structure modeling: Sensitivity to underparameterized model misspecification. Psychological Methods, 3(4), 424–453. 10.1037/1082-989X.3.4.424

[bjep12504-bib-0019] Hughes, K. , & Coplan, R. J. (2010). Exploring processes linking shyness and academic achievement in childhood. School Psychology Quarterly, 25(4), 213–222. 10.1037/a0022070

[bjep12504-bib-0020] Kalutskaya, I. N. , Archbell, K. A. , Moritz Rudasill, K. , & Coplan, R. J. (2015). Shy children in the classroom: From research to educational practice. Translational Issues in Psychological Science, 1(2), 149–157. 10.1037/tps0000024

[bjep12504-bib-0021] Kling, K. C. , Hyde, J. S. , Showers, C. J. , & Buswell, B. N. (1999). Gender differences in self‐esteem: A meta‐analysis. Psychological Bulletin, 125(4), 470–500. 10.1037/0033-2909.125.4.470 10414226

[bjep12504-bib-0022] Korpershoek, H. , Canrinus, E. T. , Fokkens‐Bruinsma, M. , & de Boer, H. (2020). The relationships between school belonging and students’ motivational, social‐emotional, behavioural, and academic outcomes in secondary education: a meta‐analytic review. Research Papers in Education, 35(6), 641–680. 10.1080/02671522.2019.1615116

[bjep12504-bib-0023] Kurdek, L. A. , & Sinclair, R. J. (2000). Psychological, family, and peer predictors of academic outcomes in first‐through fifth‐grade children. Journal of Educational Psychology, 92(3), 449–457. 10.1037/0022-0663.92.3.449

[bjep12504-bib-0024] Marino, C. , Santinello, M. , Lenzi, M. , Santoro, P. , Bergamin, M. , Gaboardi, M. , Calcagnì, A. , Altoè, G. , & Perkins, D. D. (2020). Can mentoring promote self‐esteem and school connectedness? An evaluation of the mentor‐UP project. Psychosocial Intervention, 29(1), 1–8. 10.5093/pi2019a13

[bjep12504-bib-0026] Muthén, L. K. , & Muthén, B. (2017). *M*plus user’s guide: Statistical analysis with latent variables, user’s guide. Muthén & Muthén.

[bjep12504-bib-0027] Pekrun, R. , Goetz, T. , Titz, W. , & Perry, R. P. (2002). Academic emotions in students’ self‐regulated learning and achievement: A program of qualitative and quantitative research. Educational Psychologist, 37(2), 91–105. 10.1207/S15326985EP3702_4

[bjep12504-bib-0028] Pierce, J. L. , Gardner, D. G. , & Crowley, C. (2016). Organization‐based self‐esteem and well‐being: Empirical examination of a spillover effect. European Journal of Work and Organizational Psychology, 25(2), 181–199. 10.1080/1359432X.2015.1028377

[bjep12504-bib-0029] Pomerantz, E. M. , Altermatt, E. R. , & Saxon, J. L. (2002). Making the grade but feeling distressed: Gender differences in academic performance and internal distress. Journal of Educational Psychology, 94(2), 396–404. 10.1037/0022-0663.94.2.396

[bjep12504-bib-0030] Rosenberg, M. , Schooler, C. , Schoenbach, C. , & Rosenberg, F. (1995). Global self‐esteem and specific self‐esteem: Different concepts, different outcomes. American Sociological Review, 60(1), 141–156. 10.2307/2096350

[bjep12504-bib-0031] Rubin, K. H. , & Asendorpf, J. (1993). Social withdrawal, inhibition and shyness in childhood. Lawrence Erlbaum.

[bjep12504-bib-0047] Rubin, K. H. , & Barstead, M. G. (2014). Gender differences in child and adolescent social withdrawal: A commentary. Sex Roles, 70(7), 274–284. 10.1007/s11199-014-0357-9 25709144 PMC4335803

[bjep12504-bib-0032] Rubin, K. H. , Burgess, K. B. , Kennedy, A. E. , & Stewart, S. L. (2003). Social withdrawal in childhood. In E. J. Mash , & R. A. Barkley (Eds.), Child psychopathology (pp. 372–406). Guilford Press.

[bjep12504-bib-0033] Rubin, K. H. , & Chronis‐Tuscano, A. (2021). Perspectives on social withdrawal in childhood: Past, present, and prospects. Child Development Perspectives, 15(3), 160–167. 10.1111/cdep.12417 34434251 PMC8382207

[bjep12504-bib-0050] Rubin, K. H. , Coplan, R. J. , Bowker, J. C. , & Menzer, M. (2014). Social withdrawal and shyness. In P. K. Smith , & C. H. Hart (Eds.), The wiley blackwell handbook of childhood social development (pp. 434–452). Wiley Blackwell.

[bjep12504-bib-0034] Rudasill, K. M. , Prokasky, A. , Tu, X. , Frohn, S. , Sirota, K. , & Molfese, V. J. (2014). Parent vs. teacher ratings of children’s shyness as predictors of language and attention skills. Learning and Individual Differences, 34, 57–62. 10.1016/j.lindif.2014.05.008

[bjep12504-bib-0035] Satorra, A. , & Bentler, P. M. (2001). A scaled difference chi‐square test statistic for moment structure analysis. Psychometrika, 66(4), 507–514. 10.1007/BF02296192 PMC2905175 20640194

[bjep12504-bib-0036] Steinsbekk, S. , & Wichstrøm, L. (2018). Cohort Profile: The Trondheim Early Secure Study (TESS) ‐ A study of mental health, psychosocial development and health behaviour from preschool to adolescence. International Journal of Epidemiology, 47(5), 1401–1401i. 10.1093/ije/dyy190 30215720

[bjep12504-bib-0037] Stenseng, F. , Belsky, J. , Skalicka, V. , & Wichstrøm, L. (2014). Preschool social exclusion, aggression, and cooperation: A longitudinal evaluation of the need‐to‐belong and the social‐reconnection hypotheses. Personality and Social Psychology Bulletin, 40(12), 1637–1647. 10.1177/0146167214554591 25304257

[bjep12504-bib-0038] Stenseng, F. , Belsky, J. , Skalicka, V. , & Wichstrøm, L. (2015). Social exclusion predicts impaired self‐regulation: A 2‐year longitudinal panel study including the transition from preschool to school. Journal of Personality, 83(2), 212–220. 10.1111/jopy.12096 24635533

[bjep12504-bib-0039] Stenseng, F. , Belsky, J. , Skalicka, V. , & Wichstrøm, L. (2016). Peer rejection and attention deficit hyperactivity disorder symptoms: Reciprocal relations through ages 4, 6, and 8. Child Development, 87(2), 365–373. 10.1111/cdev.12471 26671073

[bjep12504-bib-0040] Stenseng, F. , & Dalskau, L. H. (2010). Passion, self‐esteem, and the role of comparative performance evaluation. Journal of Sport and Exercise Psychology, 32(6), 881–894. 10.1123/jsep.32.6.881 21282843

[bjep12504-bib-0041] von Soest, T. , Wichstrøm, L. , & Kvalem, I. L. (2016). The development of global and domain‐specific self‐esteem from age 13 to 31. Journal of Personality and Social Psychology, 110(4), 592–608. 10.1037/pspp0000060 26167796

[bjep12504-bib-0042] Wentzel, K. R. (1991). Relations between social competence and academic achievement in early adolescence. Child Development, 62(5), 1066–1078. 10.2307/1131152 1756656

[bjep12504-bib-0043] Wentzel, K. R. , & Caldwell, K. (1997). Friendships, peer acceptance, and group membership: Relations to academic achievement in middle school. Child Development, 68(6), 1198–1209. 10.2307/1132301 9418234

[bjep12504-bib-0044] Wu, A. F. W. , Catmur, C. , Wong, P. W. , & Lau, J. Y. (2020). The presence, characteristics and correlates of pathological social withdrawal in Taiwan: An online survey. International Journal of Social Psychiatry, 66(1), 84–92. 10.1177/0020764019882724 31647367

